# Validity and Reliability of the Posturographic Outcomes of a Portable Balance Board

**DOI:** 10.3390/s25051309

**Published:** 2025-02-21

**Authors:** Patricia Meier, Maité Calisti, Inge Werner, Daniel Debertin, Lukas Mayer-Suess, Michael Knoflach, Peter Federolf

**Affiliations:** 1 Department of Neurology, Medical University of Innsbruck, 6020 Innsbruck, Austria; lukas.mayer@i-med.ac.at (L.M.-S.); michael.knoflach@i-med.ac.at (M.K.); 2 VASCage, Centre on Clinical Stroke Research, 6020 Innsbruck, Austria; 3 Department of Sports Science, University of Innsbruck, 6020 Innsbruck, Austria; maite.calisti@uibk.ac.at (M.C.); inge.werner@uibk.ac.at (I.W.); daniel.debertin@uibk.ac.at (D.D.); peter.federolf@uibk.ac.at (P.F.)

**Keywords:** postural control, force plate, balance board, balance, balance assessment, repeated measurements, reliability, validity, healthy participants, stroke

## Abstract

In view of the frequent use of the portable balance board (Tymo^®^, Tyromotion, Graz, Austria) in neurorehabilitation, the objective of this study is to assess its posturographic validity and reliability in comparison to a floor-embedded force platform (AMTI, Watertown, MA, USA), as well as its clinical validity in stroke patients. To evaluate posturographic validity, thirty healthy participants were tested in four different measurement conditions (M1–M4: standing with/without a balance pad, eyes open/closed) while simultaneously measuring with both devices placed on top of each other. Clinical validity is obtained by correlating stroke patients’ Tymo^®^ outcome variables (path length and ellipse) of M1–M4 with Berg Balance Scale (BBS) results. Reliability is determined by correlating repeated measurements. The Center of pressure (COP) paths of both force plates show a very strong correlation. The repeated measurement reliability of both force plates is comparable. Tymo^®^ outcome variables exhibit moderate to strong correlation with the BBS and demonstrated repeated measurement reliability analogous to healthy participants. Therefore, the Tymo^®^ device demonstrates measurement accuracy comparable to a gold-standard device while offering the advantages of portability and user-friendly software. Tymo^®^ is suitable for assessing balance in stroke patients, though protocol adaptations and averaging repeated measurements are recommended to enhance feasibility and reliability.

## 1. Introduction

Center of pressure (COP) trajectories obtained from floor-embedded laboratory force plates are regarded as the gold standard for the analysis of balance performance [1]. However, portable plates are a valuable tool in hospital settings where floor-embedded force plates are unavailable or where patients have reduced mobility, such as patients with stroke, Parkinson’s disease, or other neurological conditions. Some of the commercially available portable force plates are far more cost-effective and end-user-friendly than floor-embedded, gait lab-grade gold-standard force plates. However, they may have inherent limitations such as a low sample rate, inconsistent sampling intervals, and occasional signal losses when connected via Bluetooth, or other issues. Nevertheless, portable devices have demonstrated sufficient accuracy for quantifying COP trajectories during clinical balance assessments [1,2,3,4,5,6,7,8,9]. An assessment of the strengths and limitations, as well as the validity of outcome measures of these devices in comparison to the laboratory standard, is, therefore, appropriate.

The current study assessed the CE-certified portable balance board Tymo^®^ by Tyromotion, AT, which is used in clinical applications for the treatment and assessment of orthopedic and neurological patients. Furthermore, Tymo^®^ has already been used in various rehabilitation studies for the training and assessment of balance in patients with cerebral palsy [10], Parkinson’s disease [11,12], stroke [13,14], or hip-fracture patients [15]. However, to the best of our knowledge, a detailed assessment of its strengths, limitations, and validity in measurement accuracy in comparison to a laboratory-grade force platform is not yet available.

Therefore, the aim of the current study was to evaluate the validity and reliability of the Tymo^®^ balance board for assessing postural control in healthy participants by assessing (a) the correlation between COP trajectories of Tymo^®^ and a laboratory-grade force platform (AMTI), Bland–Altman plots for the stabilometric outcome variables COP-ellipse area and COP-path length, and (b) Tymo^®^’s repeated measurement reliability using intra-class correlations (ICCs). Considering the use of the device in a neurological population, the study additionally evaluates the Tymo^®^ balance board for assessing postural control in stroke patients by assessing (c) the correlation between Tymo^®^ Balance Test outcomes and the Berg Balance Scale (BBS), and (d) Tymo^®^’s repeated measurement reliability in stroke patients using intra-class correlations (ICCs).

## 2. Materials and Methods

### 2.1. Participants and Study Design

A convenience sample of 30 male and female participants volunteered in this validation study. Two age groups were recruited as follows: 15 young participants (age 20–40) and 15 elderly participants (>60 years) (Table 1). The elderly participants were included if they were able to walk independently without an assistive device. The study was conducted between 19 October 2023, to 17 November 2023, at the Department of Sport Science of the University of Innsbruck, Austria. The study had been approved by the local ethics board of the University of Innsbruck, Austria (certificate 72/2023). All the procedures were conducted in accordance with the ethical principles set down in the Declaration of Helsinki and all the participants provided written informed consent prior to testing.

Data for the validation and reliability testing of Tymo^®^ in stroke patients was obtained from a randomized controlled trial in acute stroke patients with ataxic symptoms (GLAAS II Trial) [16]. The Tymo^®^ Balance Test was used as a posturographic outcome at baseline (T0, which was just a few days post-stroke), and after four weeks of treatment (T1). The GLAAS II trial employed an experimental protocol analogous to that of the present study. However, the Tymo^®^ Balance Test was only repeated twice at each time point (T0 and T1) according to the RCT’s CRF compared to three times in our test setting with healthy volunteers (Section 2.2), where we could justify three repetitions in terms of a good health condition while accounting for better reliability.

### 2.2. Experimental Protocol

**Figure 1:**
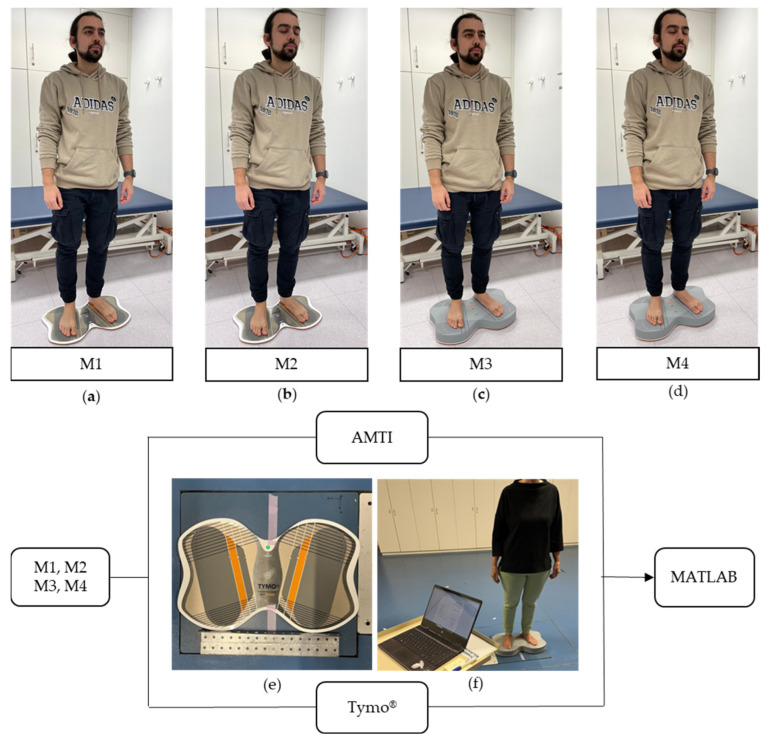
Validation test setting: (**a**–**d**) Measurement M1 to M4. (**e**) The Tymo^®^ balance board is placed on top of the AMTI force plate. Markings and a reference tool ensure the same placement of the plate at each measurement. (**f**) A healthy participant during the simultaneous recording of both devices in measurement condition M3/M4 (standing on a balance pad with eyes open/closed).

In one visit, four distinct measurements were recorded for each participant according to the Tymo^®^ Balance Test for each volunteer (Figure 1):

- M1—standing with eyes open;
- M2—standing with eyes closed;
- M3—standing on a balance pad with eyes open;
- M4—standing on a balance pad with eyes closed.

Breaks between M1 to M4 were short and at the discretion of the participant. Then, after a resting period of 5 min, measurements M1–M4 were repeated, followed by a third repetition after another 5 min rest (3 trials/participant). Anthropometric and health-related data were self-reported by the participants. Furthermore, the BBS [17] was employed to assess each participant’s balance based on a well-established clinical tool. The BBS comprises 14 tasks, including sitting, standing, reaching, and turning scored on Likert scales ranging from 0 to 4, reflecting the patient’s performance. A score of 0 indicates an inability to perform the task, while the highest possible score of the BBS is an accumulated 56 points [17].

### 2.3. Instrumentation

The Tymo^®^ balance board (Tyromotion, AT) is a commonly employed rehabilitation tool for orthopedic and neurological patients, facilitating balance training. It is a portable lightweight plate that can be connected to any computer equipped with the TyroS software 6.1 (Tyromotion, AT). It is supplied with an additional soft overlay pad and magnetic balance accessories for dynamic applications. A new added function is the “Balance Test/Posturography” to quantify balance and posture regulation in an upright stance. Different global descriptors of postural stability, e.g., the center of pressure (COP), COP path length, COP velocity, and body sway, are obtained by four force sensors integrated at each corner of the device. Given the frequent use of Tymo^®^ in clinical practice, it is of great importance to evaluate the results of the “Balance Test” obtained by this device.

Therefore, Tymo^®^ is validated against a state-of-the-art floor-embedded reference force plate (AMTI, Watertown, MA, USA) in its posturography function (“Balance Test”). It is tested for its reliability in repeated measurements in the same population. Data are obtained by simultaneously measuring with both force plates, placed on top of each other. Markings on Tymo^®^ ensure the same foot placement at each measurement (Figure 1a). The AMTI force plate recorded the data at a sampling frequency of 1000 Hz via the Vicon Nexus 2.15 software and the Tymo^®^ balance board recorded the data at a predefined measuring frequency at 50 Hz.

### 2.4. Data Processing

Data analysis was performed using a custom-written MATLAB script (The MathWorks, Version R2023a, Natick, MA, USA). For the Tymo^®^ balance board, the COP coordinates in medial–lateral (x) and anterior–posterior (y) directions were calculated from the raw data of the four included force sensors equivalent to the TyroS software (Tyromotion, AT) algorithm. For the AMTI force plate, the directly received COP coordinates were smoothed using a third-order low-pass Butterworth filter with a cut-off frequency of 8 Hz and down-sampled to the actual sampling rate of the corresponding Tymo^®^ recording, which slightly varied around 50 Hz across trials. The time series of the COP coordinates of both devices were normalized by subtracting their mean. Finally, the signals were time-synchronized by using cross-correlation. The resultant medial–lateral and anterior–posterior COP trajectories were then analyzed statistically.

Furthermore, for each trial, based on the COP output from AMTI (at the actual sampling rate), the path length and confidence ellipse area were calculated. The path length results from the following:

$${COP}_{length}=\sum_{i=1}^{n-1}\sqrt{{\left(x_{i+1}-x_{i}\right)}^{2}{+(y_{i+1}-y_{i})}^{2}}$$

with *n* being the number of observations. The area of the confidence ellipse, i.e., the ellipse that covers the COP mean with a 95% probability, was determined according to Schubert and Kirchner [18]. For the Tymo^®^ device, data concerning path length and ellipse were generated by the TyroS software.

### 2.5. Missing Data

For the analysis of the results, we excluded the M4 data from one healthy participant across all three trials, as the participant was unable to complete the task. In the first trial, we had to exclude M2 data once and M3 data twice because we obtained only incomplete data with Tymo^®^. Consequently, the data from the second trial was used for the Bland–Altman plots, as it showed no indications of learning or fatigue effects and also contained the most complete data set.

In stroke patients, missing values occurred due to an inability to perform the task, primarily for measurement M3 and M4 at baseline (T0), or due to dropout at T1, and these values were subsequently excluded.

### 2.6. Data Analysis

Statistical analysis was performed using the IBM SPSS software, release 29.0 (IBM Corporation, Armonk, NY, USA).

For the cross-correlation of the time series of the COP, we had to down-sample the AMTI data (1000 Hz) to the frequency of Tymo^®^ (50 Hz). Further, for the calculation of the variables (path length and ellipse), we did not down-sample the AMTI data, as a higher sampling rate could be a potential benefit in terms of measurement accuracy. The Pearson correlation coefficient (r) was used to calculate the correlation of the center of pressure trajectories (i.e., medial–lateral and anterior–posterior) of AMTI (down-sampled) and Tymo^®^. Bland–Altman plots were created to facilitate a comparison of the output variables (i.e., path length and ellipse) of Tymo^®^ and AMTI (actual sampling rate). To validate the clinical performance in stroke patients, the Spearman rank correlation coefficient (rs) was employed to calculate the correlation between the Tymo^®^ output variables and the BBS Score (ordinal) as the gold standard for assessing balance [19]. The BBS scores and M1-M4 values obtained four weeks after acute stroke (T1), where most of the patients were able to complete all the balance test tasks (M1–M4) yet exhibited a notable impairment in balance (according to BBS), were used for the analysis. Measurements with missing values were excluded. A coefficient above 0.90 is considered a very strong correlation, between 0.70 and 0.89 is a strong correlation, between 0.4 and 0.69 is a moderate correlation, between 0.10 and 0.39 is a weak correlation, and below 0.10 is considered a negligible correlation [20]. The two-way mixed Intraclass Correlation Coefficient (ICC 3,k; two-way-mixed; absolute agreement) was calculated with a 95% confidence interval (ICC95) to analyze the reliability of repeated measurements in healthy participants and stroke patients. Values greater than 0.90 are considered as excellent reliability, values between 0.75 and 0.9 as good, values between 0.5 and 0.75 as moderate, and values <0.5 as poor [21].

## 3. Results

### 3.1. Between-Device Comparison

**Figure 2:**
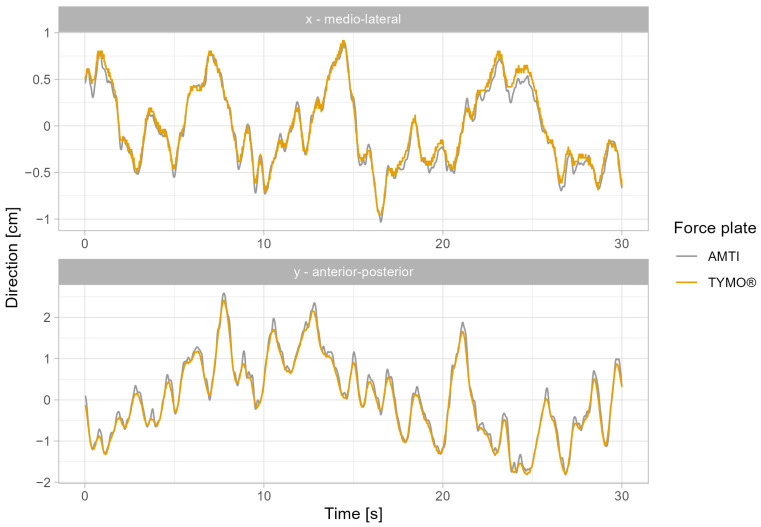
Example of the medial–lateral (x) and anterior–posterior (y) center of pressure trajectories between the AMTI force plate (blue) and Tymo^®^ (orange) for measurement condition M4 (eyes closed and balance pad) in a young participant.

**Figure 3:**
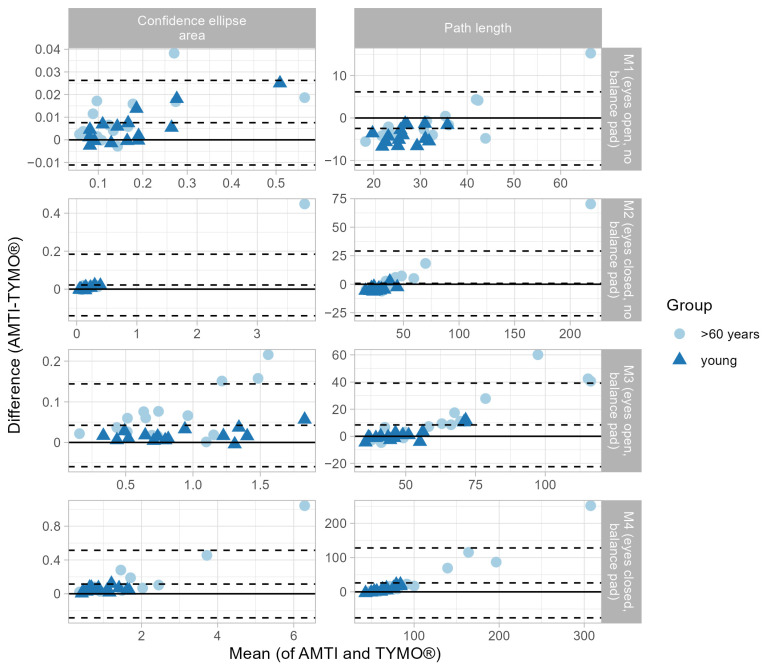
Bland–Altman plots comparing the results obtained with both devices in the four different measurement conditions of the balance test (M1–M4) for the young (dark blue pyramid) and elderly (light blue circle) participants for the output variables: ellipse in cm^2^; path length in cm.

The validity of the COP path of the medial–lateral (x) and anterior–posterior (y) directions in different measurement conditions (M1–M4) between the AMTI force plate and Tymo^®^ show a very strong correlation (Table 2), indicating the measurement accuracy of Tymo^®^ close to an established force plate. An example of the COPx and COPy shifts between AMTI and Tymo^®^ during the 30 s measurement in the M4 measurement is illustrated in Figure 2. These results are confirmed by Bland–Altman plots where 97% of the data points lie within the acceptable range in measurement conditions M1, M2, and M4. Only for measurement condition M3 the agreement was slightly lower than 95% (Figure 3). Outliers in M3 occurred at the extremities of measurements in a few individuals who showed larger postural sway. This could be explained by technical limitations like low resolution and inconsistencies in the sampling rate. Another explanation could be by clinical means, as outliers cannot be shown in M4, where the variability of the test outcomes among the participants was greater, enhancing standard deviation and therefore, upper and lower limits.

### 3.2. Repeated-Measurement Reliability

The ICC (3,1; two-way mixed; absolute agreement) for Tymo^®^ showed mostly moderate reliability for repeated measurements in the young participants and good reliability for the elderly participants (Table 3). These results are in line with the gold standard measurement device (AMTI; Table 4), showing that multiple factors other than measurement device accuracy influence balance performance.

### 3.3. Validation for Stroke Patients

When comparing the Tymo^®^ Balance Test and BBS outcomes obtained from stroke patients, a moderate to strong correlation (Spearman) was observed between BBS and path length in measurement condition M1 (r_S_ = 0.73), M2 (r_S_ = 0.60), and M3 (r_S_ = 0.60); however, only a weak correlation was found in M4 (r_S_ = 0.31). A moderate correlation was observed between BBS and the output variable ellipse for M1 (r_S_ = 0.60), M2 (r_S_ = 0.60), and M3 (r_S_ = 0.64) of the balance test, except for measurement condition M4, again showing a weak correlation (r_S_ = 0.35). Despite the most challenging measurement condition M4 (standing on a balance pad with closed eyes), these findings suggest that the balance test is a suitable tool for assessing balance in individuals who have suffered a stroke.

### 3.4. Repeated-Measurement Reliability for Stroke Patients

Using Tymo^®^ in stroke patients, the ICC for repeated measurements of the output variable path length demonstrated good reliability in M2 and M3, and moderate reliability in M1 and M4 in single measures. For the average measures, excellent reliability was shown in M2 and M3, and good reliability was observed in measurement conditions M1 and M4. When analyzing the ellipse variable, good reliability was found in single measures for M1 and M2, while M4 exhibited moderate and M3 poor reliability. For average measures, excellent reliability was seen in M1 and M2, and moderate reliability was observed in M3 and M4 (Table 5).

## 4. Discussion

The aim of this study was to evaluate the validity and reliability of the Tymo^®^ balance board for assessing postural control in healthy participants and stroke patients. Key assessments in healthy participants included the following: (a) the correlation between COP trajectories of Tymo^®^ and the AMTI force plate, Bland–Altman plots for COP path length and COP ellipse area, and (b) Tymo^®^’s reliability in repeated measurements. Further, in stroke patients, the study evaluated (c) the correlation between Tymo^®^ and BBS outcomes, and (d) Tymo^®^’s repeated measurement reliability.

**COPx and COPy path:** The results of the current study show a strong correlation between the Tymo^®^ balance board and the AMTI force plate regarding the validity of the COP path across both medial–lateral and anterior–posterior directions, indicating that Tymo^®^ provides COP path lengths comparable to the laboratory floor-embedded “gold standard”. These results are in line with the results of comparable portable devices [22,23]. Some shifts between the paths of the two devices were observed. A possible explanation could be the different sampling rates of the devices. The AMTI force plate typically operates at a higher sampling rate, which can capture rapid shifts in balance more effectively than some portable systems.

**Bland–Altman Plots (outcome variables):** A total of 97% of the data points lie within the acceptable range in measurement conditions M1, M2, and M4. Only for measurement condition M3 the agreement was lower than 95%. Tymo^®^ generally obtained smaller values of outcome variables than AMTI. This could be explained by technical limitations like low resolution [3] and inconsistencies in the sampling rate (48–50 Hz). Outliers occurred at the extremities of measurements in individuals who showed larger postural sway [3].

**Tymo^®^’s repeated measurement reliability in healthy participants:** Despite strong correlations in the COP measurements, differences in ICC values are evident. This is consistent with the nature of balance test outcomes, which tend to exhibit high variability across repeated measures due to the inherently fluctuating nature of balance [24]. The observation that these results are comparable to those obtained with a reliable floor-embedded force plate indicates that the lower ICC values are primarily influenced by clinical factors rather than measurement inconsistencies of the device. Among the older healthy participants, higher correlations were observed, likely due to the greater overall variability in their balance outcomes. This trend is also reflected in the stroke patient population, further supporting the influence of clinical variability on the observed results.

**Clinical validation in stroke patients:** A moderate to strong correlation was observed between the BBS and the Tymo^®^ output variables (path length over ellipse) for measurement conditions M1 to M3, but only a poor correlation for M4 for both outcome variables. These findings are comparable to the results obtained with other devices [25]. The poor correlation between the BBS and measurement condition M4 (balance pad and closed eyes) could be due to the lack of relatable tasks in BBS (either reduced support surface or eyes closed). Another explanation would be that Tymo output variables are more sensitive to changes due to their metric nature. Summing up, these outcomes suggest that the Tymo^®^ Balance Test is a suitable tool for assessing balance in stroke patients.

**Tymo^®^’s repeated measurement reliability in stroke patients:** The ICC for repeated measurements showed poor to good reliability in both output variables across different measurement conditions (M1–M4) for single measures (ICC 3,1). These results are in line with those obtained from our healthy participants. In the average measures (ICC 3,k), mainly good to excellent reliability was observed, while M3 and M4 still exhibited moderate reliability when analyzing the outcome variable ellipse. Based on the results, the authors recommend two trials per test session and the use of average measures to enhance the reliability of repeated measurements and therefore, the accuracy of test results to follow-up on patients. Additionally, for (sub-)acute stroke patients, measurement conditions M1 and M2 (no balance pad) are more suitable than M3 and M4 (balance pad), as most of the (sub-)acute stroke patients were not able to conduct the balance pad tasks (M3 and M4) due to severe ataxia and therefore, balance impairment. M3 and M4 measurements can be challenging even for healthy participants, as balance decreases with age, leading to difficulty standing on uneven surfaces or with closed eyes. Regarding fatigue, it would also be beneficial to reduce the amount of measurement conditions when increasing repetitions. A test scheme for Tymo^®^ for patients with severe balance impairment in an acute care setting could, therefore, be as follows: 1. trial1-M1, 2. trial1-M2, 3. trial2-M1, and 4. trial2 M2.

**Limitations:** This study has several limitations that should be considered. First, the validation of the AMIT force plate and the Tymo^®^ could not be conducted with patients. However, the inclusion of the elderly participants was intended to include greater variability in the COP and to provide patient-like conditions. Second, despite the best efforts to position the Tymo^®^ accurately on the AMTI force plate during each measurement, minor placement errors may still have occurred. Third, we were unable to synchronize the two devices in real time, and this required the data to be aligned later with a cross-correlation. Finally, the measurements (M1 to M4) were not randomized due to the constraints of the Tymo^®^ measurement software, which follows a predefined procedure and does not allow for switching between these measurements.

## 5. Conclusions

Tymo^®^’s measurement accuracy is comparable to a gold standard measurement device (AMTI), yet the board is portable, and the software is easy to use. Clinical validation was obtained by correlation with the BBS in stroke patients. Compared to the BBS, the Tymo^®^ Balance Test can be conducted more easily and faster and does not suffer from ceiling effects. However, the software measurement protocol should be adapted for (sub-)acute patients with severe balance impairment. The TYMO^®^ Balance Test should only include measurement conditions M1 and M2, as M3 and M4 measurement, where people are standing on a balance pad with their eyes closed, can be a challenging task even for healthy participants, as balance decreases with age, leading to difficulties in standing on uneven surfaces or with closed eyes. Furthermore, measurements should be repeated twice per session and average measures should be used to enhance reliability for repeated measurements. The lower ICC values for single measures are consistent with the gold standard measurement device, indicating that multiple factors beyond the accuracy of the measurement device influence balance performance, thereby making it challenging to obtain reproducible results at each measurement.

## Figures and Tables

**Table 1:** Descriptives of participants. Values are represented as mean (±standard deviation) for continuous data (age, height, and weight), as number (percent of total, %) for counted and nominal data (gender and past diseases), and as median (interquartile range) for ordinal data (BBS).

	Young (n = 15)	Elderly (>60a; n = 15)
Age (years)	30.13 (±4.22)	68.93 (±7.61)
Height (m)	1.73 (±0.09)	1.69 (±0.73)
Weight (kg)	65.68 (±8.97)	68.73 (±12.74)
Gender	m = 7 (23.3%); f = 8 (26.7%)	m = 8 (26.7%); f = 7 (23.3%)
Past diseases	Yes = 4 (13.3%)	Yes = 11 (36.7%)
	No = 11 (36.7%)	No = 4 (13.3%)
Berg Balance Scale (BBS)	56 (0.0)	56 (0.5)

**Table 2:** Validity of COP path of the medial–lateral (x) and anterior–posterior (y) directions between the AMTI force plate and Tymo^®^ in the young and elderly participants in different measurement conditions (M1—eyes open; M2—eyes closed; M3 eyes opened and balance pad; M4—eyes closed and balance pad). Values represent mean correlation coefficients (Pearson) with standard deviation and range (minimum–maximum) in brackets.

	Young	Elderly (>60a)
M1 X	0.98 (0.01; 0.94–1.00)	0.98 (0.02; 0.92–1.00)
Y	0.99 (0.00; 0.99–1.00)	0.99 (0.01; 0.97–1.00)
M2 X	0.97 (0.02; 0.91–1.00)	0.97 (0.02; 0.92–1.00)
Y	0.99 (0.00; 0.99–1.00)	0.99 (0.01; 0.97–1.00)
M3 X	0.99 (0.00; 0.98–1.00)	0.99 (0.00; 0.97–1.00)
Y	1.00 (0.00; 0.99–1.00)	0.98 (0.01; 0.94–1.00)
M4 X	0.99 (0.00; 0.98–1.00)	0.99 (0.01; 0.97–1.00)
Y	0.99 (0.01; 0.98–1.00)	0.98 (0.02; 0.91–1.00)

**Table 3:** Intraclass Correlation Coefficient (ICC 3,1; two-way-mixed; absolute agreement) for 3 repeated measurements with the Tymo^®^ balance board in the young (n = 15) and elderly (n = 15) participants in different measurement conditions (M1—eyes open; M2—eyes closed; M3—eyes opened and balance pad; M4—eyes closed and balance pad). Values are ICC (95% confidence interval).

	Young	Elderly (>60a)
Path length M1	0.63 (0.35–0.84)	0.86 (0.71–0.95)
M2	0.64 (0.36–0.84)	0.94 (0.86–0.98)
M3	0.52 (0.22–0.78)	0.84 (0.67–0.94)
M4	0.43 (0.13–0.72)	0.81 (0.60–0.93)
Ellipse M1	0.47 (0.17–0.75)	0.50 (0.19–0.77)
M2	0.62 (0.34–0.84)	0.87 (0.71–0.95)
M3	0.51 (0.13–0.77)	0.73 (0.50–0.89)
M4	0.43 (0.34–0.84)	0.26 (0.05–0.62)

**Table 4:** Intraclass Correlation Coefficient (ICC 3,1; two-way-mixed; absolute agreement) for 3 repeated measurements with the AMTI force plate in the young (n = 15) and elderly (n = 15) participants in different measurement conditions (M1—eyes open; M2—eyes closed; M3—eyes opened and balance pad; M4—eyes closed and balance pad). Values are ICC (95% confidence interval).

	Young	Elderly (>60a)
Path length M1	0.73 (0.49–0.89)	0.92 (0.83–0.97)
M2	0.72 (0.47–0.88)	0.97 (0.92–0.99)
M3	0.62 (0.32–0.84)	0.88 (0.73–0.96)
M4	0.52 (0.22–0.78)	0.84 (0.66–0.94)
Ellipse M1	0.50 (0.20–0.77)	0.51 (0.21–0.78)
M2	0.65 (0.38–0.85)	0.88 (0.73–0.95)
M3	0.50 (0.17–0.78)	0.78 (0.56–0.91)
M4	0.43 (0.10–0.73)	0.62 (0.31–0.85)

**Table 5:** Intraclass Correlation Coefficient (ICC; two-way mixed, absolute agreement) for 2 repeated measurements with the Tymo^®^ balance board in stroke patients (time point T1) in different measurement conditions (M1—eyes open; M2—eyes closed; M3—eyes opened and balance pad; M4—eyes closed and balance pad). Values are ICC (95% confidence interval).

	Single Measures (ICC 3,1)	Average Measures (ICC 3,k)
Path length M1	0.73 (0.44–0.87)	0.85 (0.61–0.93)
M2	0.83 (0.66–0.91)	0.91 (0.79–0.95)
M3	0.81 (0.67–0.90)	0.90 (0.80–0.95)
M4	0.72 (0.51–0.85)	0.84 (0.67–0.92)
Ellipse M1	0.86 (0.75–0.92)	0.93 (0.86–0.96)
M2	0.89 (0.81–0.94)	0.94 (0.89–0.97)
M3	0.45 (0.17–0.67)	0.62 (0.28–0.80)
M4	0.58 (0.30–0.77)	0.74 (0.46–0.87)

## Data Availability

The data presented in this study are available upon request from the corresponding author. The data are not publicly available due to privacy concerns.
